# Development of convolutional neural network model for diagnosing meniscus tear using magnetic resonance image

**DOI:** 10.1186/s12891-022-05468-6

**Published:** 2022-05-30

**Authors:** Hyunkwang Shin, Gyu Sang Choi, Oog-Jin Shon, Gi Beom Kim, Min Cheol Chang

**Affiliations:** 1 Department of Information and Communication Engineering, Yeungnam University, Gyeongsan-si, Republic of Korea; 2 Department of Orthopedic Surgery, Yeungnam University College of Medicine, Yeungnam University, 317-1, Daemyungdong, Namku, Daegu, 42415 Republic of Korea; 3 Department of Physical Medicine and Rehabilitation, College of Medicine, Yeungnam University, 317-1, Daemyungdong, Namku, Daegu, 42415 Republic of Korea

**Keywords:** Deep learning, Convolutional neural network, Magnetic resonance imaging, Meniscus tear, Knee

## Abstract

**Background:**

Deep learning (DL) is an advanced machine learning approach used in diverse areas, such as image analysis, bioinformatics, and natural language processing. A convolutional neural network (CNN) is a representative DL model that is advantageous for image recognition and classification. In this study, we aimed to develop a CNN to detect meniscal tears and classify tear types using coronal and sagittal magnetic resonance (MR) images of each patient.

**Methods:**

We retrospectively collected 599 cases (medial meniscus tear = 384, lateral meniscus tear = 167, and medial and lateral meniscus tear = 48) of knee MR images from patients with meniscal tears and 449 cases of knee MR images from patients without meniscal tears. To develop the DL model for evaluating the presence of meniscal tears, all the collected knee MR images of 1048 cases were used. To develop the DL model for evaluating the type of meniscal tear, 538 cases with meniscal tears (horizontal tear = 268, complex tear = 147, radial tear = 48, and longitudinal tear = 75) and 449 cases without meniscal tears were used. Additionally, a CNN algorithm was used. To measure the model’s performance, 70% of the included data were randomly assigned to the training set, and the remaining 30% were assigned to the test set.

**Results:**

The area under the curves (AUCs) of our model were 0.889, 0.817, and 0.924 for medial meniscal tears, lateral meniscal tears, and medial and lateral meniscal tears, respectively. The AUCs of the horizontal, complex, radial, and longitudinal tears were 0.761, 0.850, 0.601, and 0.858, respectively.

**Conclusion:**

Our study showed that the CNN model has the potential to be used in diagnosing the presence of meniscal tears and differentiating the types of meniscal tears.

## Background

A meniscus tear resulting from trauma or degeneration is a common cause of persistent knee pain [1]. It also results in a reduction in function, a low quality of life, and early osteoarthritis [2]. Accurate detection of meniscal tears is essential for adequate and effective treatment. In addition, based on the type of meniscal tear, the treatment options can range from conservative to surgical [3, 4]. Magnetic resonance imaging (MRI) is the most useful and accurate non-invasive diagnostic tool for the diagnosis of meniscal tears. It is typically used as the first method for evaluating suspected meniscal tears and can effectively present the location and type of meniscal tear [5]. However, the diagnostic accuracy of MRI for evaluating the presence of meniscal tears and type of tear is different between clinicians specializing in knee disease and other clinicians. A system that aids in reading a knee MRI would be of great help for clinicians to manage patients suspected of having a meniscus tear.

Machine learning (ML) is a computer algorithm that automatically learns from data without requiring explicit programming [6]. ML enables breakthroughs in several fields, such as big data analysis, image analysis, natural language processing, and bioinformatics [7–12]. In addition, the usefulness of ML in the diagnosis of various musculoskeletal disorders has been demonstrated [13–15]. The deep learning (DL) technique is an advanced ML approach. DL involves the construction of artificial neural networks using numerous hidden layers with structures and functions similar to those of the human brain [16]. The DL technique can learn unstructured and perceptual data, such as images and languages, and overcome traditional ML techniques. A convolutional neural network (CNN) is a representative DL model that is advantageous, particularly in image recognition and classification [17]. Previous studies have shown that a CNN can be useful for determining the presence of meniscal tears in knee MRI images [18–21]. A CNN model that can differentiate tear location in the anterior horn, body, and posterior horn was recently developed [21]. We assumed that the CNN could be useful for classifying tear types (horizontal, complex, radial, and longitudinal tears) in addition to detecting meniscal tears.

In this study, we developed a CNN model to diagnose meniscal tears, classify the types of meniscal tears using knee magnetic resonance (tablMR) images of each patient, and evaluate its accuracy.

## Methods

### Subjects

**Fig. 1:**
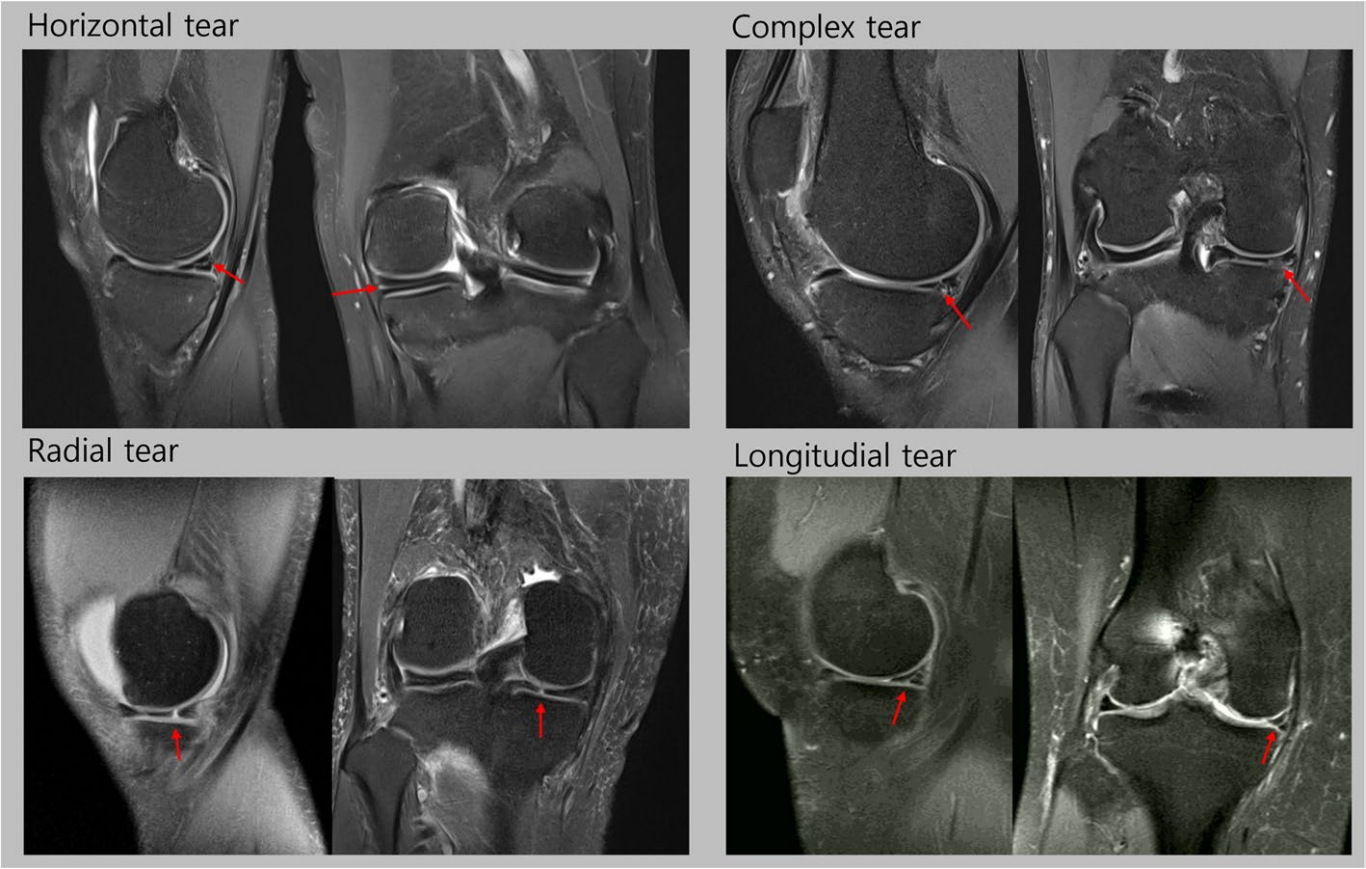
Representative magnetic resonance images of each type of meniscus tear

We retrospectively collected 599 knee MR images from patients with meniscal tears, and 449 knee MR images from patients without meniscal tears. All MR images were obtained from a single university hospital from January 2010 to December 2020 (mean age = 38.7 ± 16.5; M:F = 729:319). To develop the DL model for evaluating the presence of meniscal tears, all collected knee MR images of the 599 cases with meniscal tears (medial meniscus tear = 384, lateral meniscus tear = 167, medial and lateral meniscus tears = 48) and 449 cases without meniscal tears were used. Tear of the meniscus on MR images was independently assessed by two board-certified orthopedic knee specialists and repeated 2 weeks later. If there was a disagreement between the two experts, a third orthopedic knee specialist made the final decision on the grade. Reliabilities for all radiographic parameters were analyzed using intra-class correlation coefficients and were classified as little (correlation coefficient, ≤ 0.25), low (0.26–0.49), moderate (0.50–0.69), high (0.70–0.89), or very high (≥ 0.90) [22]. To develop a DL model for evaluating the type of meniscal tear, 538 cases with meniscal tears (horizontal tear = 268, complex tear = 147, radial tear = 48, longitudinal tear, 75) (Fig. 1) and 449 cases without meniscal tears were used. The study protocol was approved by the institutional research board of the university hospital. The Institutional Review Board waived the requirement for written informed consent because this study was performed retrospectively using anonymous data**.** The Helsinki Declaration was adhered to in this study.

### Images used for deep learning (input variables)

All MRI examinations were performed using a 1.5 T MR scanner (Philips Medical Systems, Eindhoven, Netherlands). We used fat-suppressed T2-weighted coronal and sagittal images containing the meniscus (repetition time, 2480–5000 ms; echo time, 19–25 ms; section thickness, 4 mm; NEX, 3.0; 192 × 2; matrix, 192 × 256).

### Deep learning model

This study consisted of two main components: 1) determining meniscal tears and 2) classifying tear type. In this study, we trained the model for tear detection and tear type independently.

### CNN model for meniscus tear

**Fig. 2:**
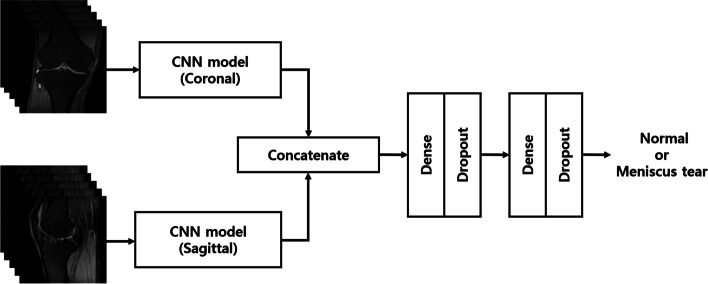
Illustration of the convolutional neural network model determining the presence of a meniscus tear. CNN: convolutional neural network

Coronal and sagittal MR images were used as inputs to determine the presence of meniscal tears, and the features of coronal and sagittal MRI images were extracted using two CNN models. The CNN model used AlexNet as the backbone, and the input size of each CNN model was s × 224 × 224 × 3 [23]. Here, s indicates the number of 2D images included in the MRI and 3 indicates the number of RGB color channels. Each CNN model consisted of five convolutional layers and a global average pooling layer. The feature maps generated in each model are concatenated and delivered to the fully connected layer. The fully connected layer of the model consists of two layers. These two layers contained a dropout layer and used a sigmoid function to classify meniscal tears. Figure 2 illustrates the CNN model used to identify the meniscal tears. The detailed architecture of the CNN model is shown in Table 1.

**Table 1:** Architecture of the convolutional neural network model for determining the presence of a meniscus tear

Layer	Kernel size (stride, padding)	Feature size	
		Coronal CNN model	Sagittal CNN model
Input	–	s × 224 × 224 × 3	s × 224 × 224 × 3
Convolution + ReLU	11 × 11 (4, 2)	s × 55 × 55 × 64	s × 55 × 55 × 64
Max pooling	3 × 3 (2, 0)	s × 27 × 27 × 64	s × 27 × 27 × 64
Convolution + ReLU	5 × 5 (1, 2)	s × 27 × 27 × 192	s × 27 × 27 × 192
Max pooling	3 × 3 (2, 0)	s × 13 × 13 × 192	s × 13 × 13 × 192
Convolution + ReLU	3 × 3 (1, 1)	s × 13 × 13 × 384	s × 13 × 13 × 384
Convolution + ReLU	3 × 3 (1, 1)	s × 13 × 13 × 256	s × 13 × 13 × 256
Convolution + ReLU	3 × 3 (1, 1)	s × 13 × 13 × 256	s × 13 × 13 × 256
Max pooling	3 × 3 (2, 0)	s × 6 × 6 × 256	s × 6 × 6 × 256
Adaptive average pooling, max value extraction	7 × 7	s × 1 × 1 × 256, 1 × 1 × 1 × 256	s × 1 × 1 × 256, 1 × 1 × 1 × 256
Concatenate	–	1 × 1 × 1 × 512	
Dens + Dropout (0.5)	–	1 × 1 × 1 × 256	
Dens + Dropout (0.3)		1 × 1 × 1 × 128	
Output + sigmoid		1	

*CNN* convolutional neural network

### CNN model for the type of meniscus tear

**Fig. 3:**
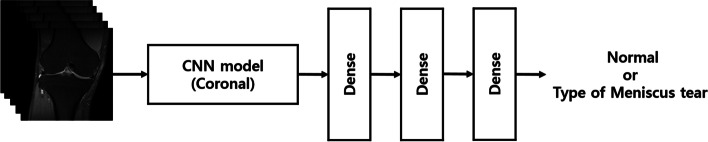
Illustration of the convolutional neural network model for determining the type of meniscus tear. CNN: convolutional neural network

Coronal MR images were used as inputs to classify the type of meniscal tear. Our CNN model extracted image features for the meniscus type using AlexNet as the backbone. The input size of this CNN model was s × 224 × 224 × 3, and the features of the meniscus image were extracted through each of the five convolutional layers. The extracted feature maps were averaged using image slices, and then transferred to a fully connected layer. The fully connected layer comprised of three layers, and the sigmoid function was used as the last activation function. Figure 3 illustrates the CNN model used to determine the type of meniscal tears. The detailed architecture of the CNN model is shown in Table 2.

**Table 2:** Architecture of the convolutional neural network model for differentiating the type of meniscus tear

Layer	Kernel size (stride, padding)	Feature size
Input	–	s × 224 × 224 × 3
Convolution + ReLU	11 × 11 (4, 2)	s × 55 × 55 × 64
Max pooling	3 × 3 (2, 0)	s × 27 × 27 × 64
Convolution + ReLU	5 × 5 (1, 2)	s × 27 × 27 × 192
Max pooling	3 × 3 (2, 0)	s × 13 × 13 × 192
Convolution + ReLU	3 × 3 (1, 1)	s × 13 × 13 × 384
Convolution + ReLU	3 × 3 (1, 1)	s × 13 × 13 × 256
Convolution + ReLU	3 × 3 (1, 1)	s × 13 × 13 × 256
Max pooling	3 × 3 (2, 0)	s × 6 × 6 × 256
Adaptive average pooling, max value extraction	7 × 7	s × 1 × 1 × 256, 1 × 1 × 1 × 256
Dens	–	1 × 1 × 1 × 128
Dens	–	1 × 1 × 1 × 64
Output + sigmoid	–	1

### Implementation details

All of our models were implemented in PyTorch version 1.7.0 and were tested on an NVIDIA GeForce RTX 2080TI. All MR images were normalized between 0 and 1 (pixel value/255). We retrained the model using the weight of the pretrained AlexNet model as the initial weight. The batch size and epoch of each model were set to 1 and 100, respectively, and the training model was optimized using the Adam optimizer method.

### Dataset

The MRI data of meniscal tears were categorized as follows: 1) To develop a model to determine the presence of meniscal tears: normal, medial meniscus, lateral meniscus, and medial and lateral meniscal tears. 2) To develop a model to differentiate between the types of meniscal tears: normal, horizontal, complex, radial, and longitudinal.

The details of the dataset configurations are presented in Tables 3 and 4. For each case, 70% of the dataset was randomly selected as the training set, whereas the remaining 30% was assigned to the test set to evaluate the model performance.

**Table 3:** Dataset of the presence of meniscal tear

Category	Training set	Testing set	Total
Medial meniscus tear (Normal/Medial meniscus tear)	583 (314/269)	250 (135/115)	833
Lateral meniscus tear (Normal/ Lateral meniscus tear)	431 (314/117)	185 (135/50)	616
Medial and lateral meniscus tear (Normal/Medial and lateral meniscus tear)	348 (314/34)	149 (135/14)	497

**Table 4:** Dataset of the type of meniscal tear

Category	Training set	Testing set	Total
Horizontal tear (Normal/Horizontal)	502 (314/188)	215 (135/80)	717
Complex tear (Normal/Complex)	417 (314/103)	179 (135/44)	596
Radial tear (Normal/Radial)	348 (314/34)	149 (135/14)	497
Longitudinal tear (Normal/Longitudinal tear)	367 (314/53)	157 (135/22)	524

### Evaluation metrics

The performance of the model was evaluated in terms of accuracy, precision, recall, sensitivity, specificity, and area under the curve (AUC). The 95% confidence interval for the AUC was calculated using the method described by DeLong et al. [24].

## Results

We evaluated our model performance and compared it with MobileNet [25]. We used the same hyper-parameters for MobileNet and our model. In addition, the fully connected layer of MobileNet was modified, as in our model.

Table 5 shows the performance of the models that were employed to identify the presence of meniscal tears. The AUCs of our model were 0.889, 0.817, and 0.924 for medial meniscal, lateral meniscal, and medial and lateral meniscal tears, respectively, with an accuracy of 85.08, 80.54, and 91.95%, respectively. Furthermore, the precisions of the medial meniscal, lateral meniscal, and medial and lateral meniscal tears were 83.93, 62.96, and 55%, respectively. The sensitivity/specificity of the medial meniscal, lateral meniscal, and medial and lateral meniscal tears were 83.19%/86.67, 68%/85.19, and 78.57%/93.33%, respectively. As compared with MobileNet, the proposed model showed improvements in the accuracy, precision, recall, sensitivity, specificity, and AUC by 20.97, 21.93, 28.32, 28.32, 14.82%, and 0.214, respectively, in identifying medial meniscus tears. Further, for lateral meniscus tears, the metrics improved by 16.22, 22.96, 4, 4, 20.75%, and 0.143, respectively, for the proposed model. The metrics associated with medial and lateral meniscus tears improved by 16.78, 34.49, 21.43, 21.43, 16.29%, and 0.273, respectively.

**Table 5:** Performance of the deep learning model for the presence of a meniscal tear

	Model	Acc	Pre	Rec	Sen	Spe	AUC (95% CI)	Time (sec)
Medial tear	MobileNet	64.11%	62%	54.87%	54.87%	71.85%	0.675 (0.608–0.742)	6.02
	Ours	85.08%	83.93%	83.19%	83.19%	86.67%	0.889 (0.845–0.933)	3.64
Lateral tear	MobileNet	64.32%	40%	64%	64%	64.44%	0.674 (0.592–0.756)	4.48
	Ours	80.54%	62.96%	68%	68%	85.19%	0.817 (0.744–0.889)	2.77
Medial and lateral tear	MobileNet	75.17%	20.51%	57.14%	57.14%	77.04%	0.651 (0.476–0.825)	3.15
	Ours	91.95%	55%	78.57%	78.57%	93.33%	0.924 (0.863–0.985)	1.88

*ACC* accuracy, *Pre* precision, *Rec* recall, *Sen* sensitivity, *Spe* specificity, *AUC* area under the curve, *CI* confidence interval

**Fig. 4:**
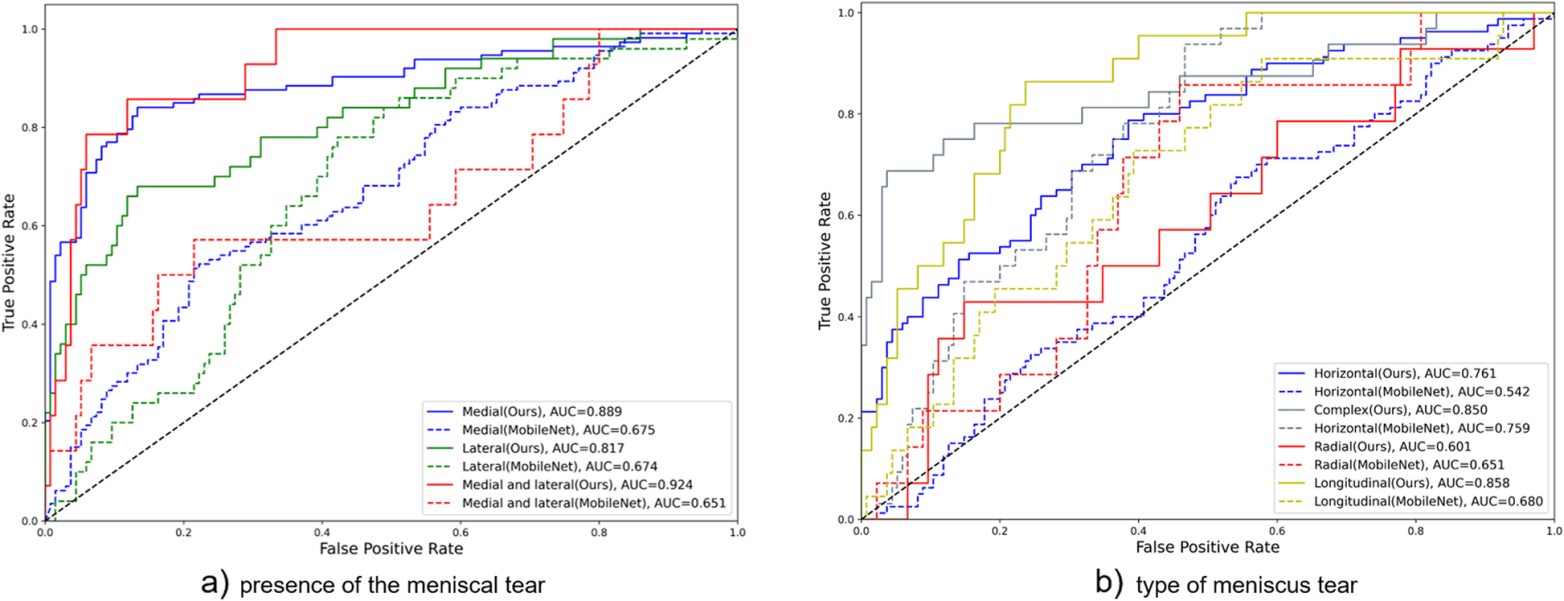
Receiver operating characteristic curve and area under the curve for the test dataset. AUC: area under the curve

Table 6 presents the performance results for the different types of meniscal tears. The AUCs of our model were 0.761, 0.85, 0.601, and 0.858 for the horizontal, complex, radial, and longitudinal tears, respectively, with an accuracy of 72.23, 91.02, 72.48, and 81.53%, respectively. Additionally, the precision of the horizontal, complex, radial, and longitudinal tears were 59.3, 81.48, 15.38, and 40.54%, respectively. The sensitivity/specificity of the horizontal, complex, radial, and longitudinal tears were 63.75%/74.07, 68.75%/96.3, 42.86%/75.56, and 68.18%/83.7%, respectively. We observed that, as compared with MobileNet, the accuracy, precision, specificity, and the AUC improved by 20.14, 17.82, 32.59%, and 0.219, respectively, for the proposed model in the case of horizontal tears. These metrics for complex tears improved by 26.95, 49.43, 35.56%, and 0.091, respectively, for the proposed model. For radial tears, the proposed model performed better than MobileNet with improvements of 27.93, 66.23, 4.46, 4.46, and 33.34% in terms of accuracy, precision, recall, sensitivity, and specificity, respectively. For longitudinal tears, the proposed model showed improvements of 15.29, 18.72, 13.63, 13.63, 15.55%, and 0.178 in terms of accuracy, precision, recall, sensitivity, specificity, and AUC, respectively. Figure 4 shows the receiver operating characteristic curve results for test dataset. The meniscal tears assessed by two orthopedic surgeons (GBK and OS) showed very high intra- and inter-observer reliabilities (Table 7).

**Table 6:** Performance of the deep learning model for the type of a meniscal tear

	Model	Acc	Pre	Rec	Sen	Spe	AUC (95% CI)	Time (sec)
Horizontal tear	MobileNet	52.09%	41.48%	70%	70%	41.48%	0.542 (0.463–0.621)	2.45
	Ours	72.23%	59.3%	63.75%	63.75%	74.07%	0.761 (0.694–0.828)	1.26
Complex tear	MobileNet	64.07%	32.05%	78.12%	78.12%	60.74%	0.759 (0.682–0.835)	1.91
	Ours	91.02%	81.48%	68.75%	68.75%	96.3%	0.850 (0.759–0.941)	1.01
Radial tear	MobileNet	63.09%	15.25%	64.29%	64.29%	62.96%	0.651 (0.517–0.785)	1.76
	Ours	72.48%	15.38%	42.86%	42.86%	75.56%	0.601 (0.433–0.768)	0.95
Longitudinal tear	MobileNet	66.24%	21.82%	54.55%	54.55%	68.15%	0.680 (0.561–0.798)	1.71
	Ours	81.53%	40.54%	68.18%	68.18%	83.7%	0.858 (0.787–0.930)	1.03

*ACC* accuracy, *Pre* precision, *Rec* recall, *Sen* sensitivity, *Spe* specificity, *AUC* area under the curve, *CI* confidence interval

**Table 7:** Intra- and inter-class correlation coefficients of the meniscal tear on magnetic resonance images

	Intra-observer	Inter-observer
Normal	0.98	0.96
Horizontal tear	0.96	0.93
Complex tear	0.91	0.90
Radial tear	0.93	0.91
Longitudinal tear	0.94	0.93

Values are presented as absolute values. The data showed almost perfect intra- and inter-observer agreement for the measured parameters [12]

## Discussion

In this study, we developed a CNN model for detecting the presence and type of meniscal tears using MR images as input data.

The AUCs for detecting the presence of tears in the medial meniscal, lateral meniscal, and both medial and lateral meniscal were 0.889, 0.817, and 0.924, respectively (Fig. 4a). Considering that an AUC ≥ 0.9, 0.9 > AUC ≥ 0.8, and 0.8 > AUC ≥ 0.7 are generally outstanding, excellent, and acceptable [26], respectively, our model trained using knee MRI as input data can be potentially applied for diagnosing meniscal tears in clinical practice. Regarding the capacity to differentiate the type of meniscal tear, the AUCs were 0.761, 0.850, 0.601, and 0.858 for horizontal, complex, radial, and longitudinal tears, respectively (Fig. 4b). In addition to radial tears, determination of the other three types of meniscal tears was acceptable.

A DL model consists of a multilayer perceptron with multiple hidden layers, or a feedforward neural network. It has a greater ability to learn the characteristics of input data in detail than traditional shallow neural networks [16]. A CNN is a representative deep learning (DL) model. It receives multiple channels of two-dimensional data as input and transforms them repeatedly using convolution and pooling operations [17]. These processes allow the extraction of valuable features from the input data. Therefore, CNNs have been used to recognize image patterns and process image data [17]. Our developed model recognized the valuable characteristics of knee MR images, identified meniscal tears, and classified the images based on the type of meniscal tear. However, our model has a low capacity for detecting and diagnosing radial meniscal tears. This could be because a small number of cases of radial tears were used to develop the DL model compared to other types of meniscal tears. In addition, the relatively small size of the lesion observed on MRI in radial tears could be attributed to the low AUC result.

To the best of our knowledge, four previous studies have evaluated the diagnostic efficacy of the DL model for detecting meniscal tears on knee MRI [18–21]. In 2018, Bien et al. developed a CNN model using 1370 cases of knee MRI (coronal, sagittal, and axial MR images; meniscus tear, 397) [18]. The AUC value for determining the presence of meniscal tears was 0.847. In 2020, Fritz et al. used a training set of 18,520 MR images, 1000 MR images for the validation set, and 1000 MR images for testing data. They developed a DCNN consisting of two 3D convolutional blocks (coronal and sagittal) to determine the presence of meniscal tears [19]. The AUC value for diagnosing medial meniscal tears was 0.882, that for lateral meniscal tears was 0.781, and that for overall meniscal tears was 0.961. Moreover, Rizk et al. used coronal and sagittal knee MR images from 11,353 examinations [20]. The AUC value for diagnosing medial meniscal tears was 0.93 and that for lateral meniscal tears was 0.84. Most recently, in 2021, Tack et al. used 2399 sagittal 3-dimensional MRI scans from the publicly available database of the Osteoarthritis Initiative [21]. The AUC values for medial meniscal tears in the anterior horn, body, and posterior horn were 0.94, 0.93, and 0.93, respectively, whereas those for lateral meniscal tears were 0.96, 0.94, and 0.91, respectively. Recent studies have reported an enhancement in the accuracy of DL models for diagnosing meniscal tears [20, 21]. This can be attributed to the large number of MRI scans required. However, previous studies did not diagnose the type of meniscal tear. Therefore, our study is the first to develop a DL model to classify meniscal tears based on knee MRI. Table 8 summarizes related work on meniscal tears.

**Table 8:** Summary of related works on meniscus tears

	Method	Advantage
Bien et al. [18]	• Each CNN models for the coronal, sagittal, and axial plan MR images is trained. The predicted results from each CNN model determine the meniscus tear through a logistic regression model.	• By utilizing the result that had been predicted for each model (coronal, sagittal, and axial models), the performance had been improved.
Fritz et al. [19]	• In the coronal and sagittal MR images, after extracting the meniscal ROI, two 3D convolution blocks are used to determine the presence of meniscal tears.	• By utilizing the 3D space information, the performance had been improved.
Rizk et al. [20]	• By using the meniscal localizer model that is organized with three convolution layers, the meniscal ROI is extracted. After, the presence of a meniscus tear is then determined through a meniscus tear detection model.	• Although, because the meniscal ROI area is extracted, the operation quantity for a model that decides the existence of the meniscal tears gets reduced, an accurate meniscal ROI area must be extracted.
Tack et al. [21]	• Regarding the 3D MR images entry, by using the U-Net Model, the area that belongs to the meniscus is extracted. And, through the ResNet encoder, presence of meniscal tears is decided.	• Although the meniscus tear model that is based on the previous 2D MR images does not consider the entire MR images volume, the model that was proposed had improved the performance by utilizing the 3D space information.

## Conclusions

In conclusion, using coronal and sagittal knee MR images, we developed a CNN model to diagnose the presence of meniscal tears and differentiated types of meniscal tears. The diagnostic accuracy is generally acceptable. Although our CNN model is limited in its low accuracy for diagnosing radial tears, we believe that our study is meaningful because it is the first to distinguish the types of meniscal tears and show the possibility that the CNN model can differentiate types of meniscal tears and detect the presence of meniscal tears. In the future, diagnostic accuracy should be increased by using a larger amount of knee MRI data.

## Data Availability

The datasets used and/or analyzed during the current study are available from the corresponding author upon reasonable request.

## Abbreviations

MRI: Magnetic resonance imaging
ML: Machine learning
DL: Deep learning
CNN: Convolutional neural network
AUC: Area under the curve
